# Effects of crop load on distribution and utilization of ^13^C and ^15^N and fruit quality for dwarf apple trees

**DOI:** 10.1038/s41598-017-14509-3

**Published:** 2017-10-26

**Authors:** Ning Ding, Qian Chen, Zhanling Zhu, Ling Peng, Shunfeng Ge, Yuanmao Jiang

**Affiliations:** State Key Laboratory of Crop Biology, College of Horticulture Science and Engineering, Shandong Agricultural University, Tai’an, Shandong 271018 China

## Abstract

In order to define the effects of fruit crop load on the distribution and utilization of carbon and nitrogen in dwarf apple trees, we conducted three crop load levels (High-crop load, 6 fruits per trunk cross-sectional area (cm^2^, TCA)), Medium-crop load (4 fruits cm^−2^ TCA), Low-crop load (2 fruits cm^−2^ TCA)) in 2014 and 2015. The results indicated that the ^15^N derived from fertilizer (Ndff) values of fruits decreased with the reduction of crop load, but the Ndff values of annual branches, leaves and roots increased. The plant ^15^N-urea utilization rates on Medium and Low-crop load were 1.12–1.35 times higher than the High-crop load. With the reduction of crop load, the distribution rate of ^13^C and ^15^N in fruits was gradually reduced, but in contrast, the distribution of ^13^C and ^15^N gradually increased in annual branches, leaves and roots. Compared with High-crop load, the Medium and Low-crop load significantly improved fruit quality p < 0.05. Hence, controlling fruit load effectively regulated the distribution of carbon and nitrogen in plants, improved the nitrogen utilization rate and fruit quality. The appropriate crop load level for mature M.26 interstocks apple orchards was deemed to be 4.0 fruits cm^−2^ TCA.

## Introduction

According to the characteristics of the output and input of plant photosynthetic products, the tissues and organs can be divided into two categories, namely “sink” and “source”. The “source” refers to the organs responsible for the production and transportation of nutrients to other organs, mainly designating the blade. The “sink” refers to the organs consuming or reserving nutrients, such as young leaves, stems, roots, flowers, fruit, seeds, etc. For fruit trees, the fruit is the main organ of the “sink”, and there is competition for nutrients in the growth and development process. Excessive amount of fruits per tree decrease fruit size and quality, consume tree reserves and reduce cold hardiness^1^. Only the balanced distribution of assimilation products in the organs of sink and source can guarantee the high economic yield. Therefore, it is of great significance to adjust the “sink-source” relationship in fruit trees for the growth and development of fruit, fruit quality and storage nutrition.

The crop load is one of the most important factors influencing the relationship of sink (fruit)-source (leaf) of fruit trees. With inappropriate crop load, the photosynthesis and storage nutrient of fruit trees were adversely influenced, which resulted in the phenomenon of biennial bearing for continuous years^2–4^. Smitha and Samach^5^ found that high crop load of fruit trees led to the weakness of tree vigor and affected the development of leaves which resulted in the fruit trees’ senescence at later growth stage^6,7^. Additionally, high crop load reduced trees storage nutrition, which significantly affected the vegetative growth and flower bud differentiation in the second year^8^.

Fruit thinning is effective in managing the relationship between vegetative and reproductive growth, which ensures high quality and yield in fruit trees by adjusting the relationship between “sink” and “source” and changing the transportation and distribution of photosynthate^9,10^. Fruit thinning has been shown successfully to overcome alternate bearing, increase nutrient accumulation, and prevent premature aging^11^. And, numerous researches have demonstrated that the proper fruit thinning can improve the average weight of fruit, improve the fruit quality and fruit commodity rate^12,13^. Meanwhile, the proper crop load can be conducive for improving the leaf photosynthesis^14,15^.

The reasonable crop load is a crucial factor of guaranteeing the tree’s growth to gain the high yield, stable production and good quality^16,17^. However, the tree’s growth and its yield are closely related to the nutrient distribution of carbon and nitrogen. The carbon nutrition directly affects the growth and structure of trees, and the output and quality of fruits^18,19^. As the essential mineral element of fruit trees, nitrogen is closely related to tree’s vegetative and reproductive growth, and has significant impact on the flower bud formation, yield and fruit quality, particularly fruit size and color^20–22^. Carbon and nitrogen metabolism are the most important metabolic pathways in plants, which is intimately related with each other^23^. The nitrogen metabolism provides enzymes and photosynthetic pigments for the carbon metabolism. The suitable nitrogen nutrition effectively improves the leaf photosynthesis and the chlorophyll. The photosynthetic rate in per unit area increased with the increase of nitrogen content, but the assimilation rate of plants decreased when the nitrogen reached to a certain value^24^. Similarly, the aboveground growth of plants can promote the absorption of nitrogen in roots. Under insufficient illumination, the activity of roots is reduced, affecting the absorption of nitrogen and the photosynthesis of leaves^25^. Thus, coordinating the reasonable distribution of carbon and nitrogen in plants is of great significance to improve their production.

During the past three decades, the system of apple cultivation in the world has undergone the profound changes. The cultivation of apple under dwarf and close planting has become the trend and direction of the apple cultivation^26–30^. M.26, the dwarf interstock, is the most widely used in dwarf apple orchards in China, which is accounting for 70% of the total cultivation area of dwarf apples^31^. In modern commercial apple orchards, dwarf apple trees posses many advantages with producing more flower buds, flowers and fruit etc., so the crop load of fruit trees is generally too high for the production, which becomes the main cause of biennial bearing. Previous research has focused on the effects of crop load on the growth of trees, the photosynthesis of leaves, and fruit yield and quality^32–37^. However, the studies on effects of the crop load on the distribution and utilization of carbon and nitrogen for the dwarf apples have not been reported. Therefore, the objectives of this study were to define the mechanism of the fruit crop load on the growth and development of dwarf apples, from the perspective of carbon/nitrogen distribution. For the concept, the effects of the different crop load treatments on the distribution and utilization of carbon and nitrogen were investigated in Red ‘Fuji’ apple on M.26 interstocks with ^13^C and ^15^N tracer technology. The results will provide scientific basis for cultivation and rational fertilization of dwarf apple trees.

## Results

### Leaf area, SPAD values, photosynthetic rate, the total nitrogen content of leaves

The leaf area, SPAD values, Pn of apple in Medium and Low-crop load treatments were significantly higher compared with the High-crop load treatment, among which the Low-crop load treatment was the highest at the fruit maturity stage in both years (Table 1). The total nitrogen content of leaves in the Medium and Low-crop load treatments were 1.24 and 1.80 times larger than that of the High-crop load treatment respectively in 2014, and 1.44 and 1.90 times in 2015. The root-shoot ratio was significantly affected by fruit crop load under the same nitrogen level, which gradually increased with fruit crop load decreasing. Compared with the High-crop load treatment, the root-shoot ratio in Medium and Low-crop load treatments increased by 12.90% and 25.81% in 2014, and 11.76% and 26.47% in 2015, respectively. The results suggested that the decline of crop load can significantly increase the leaf area, SPAD values and the total nitrogen content of leaves so as to improve the leaf Pn and delay leaf senescence. Meanwhile, the decline of crop load can significantly increase the root-shoot ratio.

**Table 1:** Effects of different crop load treatments on leaf area, SPAD, the total N content of leaves at the fruit maturity stage in 2014 and 2015.

Treatment	2014					2015				
	Leaf area (cm^2^)	SPAD (arbitrary units)	Pn (μmol·m^2^·s^−1^)	Total N content of leaves (g)	Root- shoot ratio	Leaf area (cm^2^)	SPAD (arbitrary units)	Pn (μmol·m^2^·s^−1^)	Total N content of leaves (g)	Root- shoot ratio
High-crop load (6.0 fruits cm^−2^ TCA)	27.51 ± 0.5260 c	48.36 ± 0.9341 c	10.23 ± 0.5033c	13.47 ± 0.2219 c	0.31 ± 0.0275 c	28.01 ± 0.3213 c	47.89 ± 0.4821c	9.78 ± 0.2926 c	13.28 ± 1.1077 c	0.34 ± 0.0156 c
Medium-crop load (4.0 fruits cm^−2^ TCA)	29.97 ± 0.4474 b	52.37 ± 1.115 b	11.83 ± 0.5508 b	16.76 ± 1.0372 b	0.35 ± 0.0164 b	31.34 ± 0.9209 b	53.70 ± 1.0782 b	12.30 ± 0.3603 b	19.18 ± 1.3471 b	0.38 ± 0.0392 b
Low-crop load (2.0 fruits cm^−2^ TCA)	32.84 ± 0.7759 a	55.70 ± 0.4795 a	13.50 ± 0.2646 a	24.23 ± 1.4333 a	0.39 ± 0.0275 a	34.92 ± 0.7427 a	56.33 ± 2.0324 a	14.06 ± 0.2517 a	25.27 ± 0.6875 a	0.43 ± 0.0269 a

Note: Means followed by similar letter within each column are not significantly different at the 0.05 level.

### The Ndff values of plant organs

**Figure 1:**
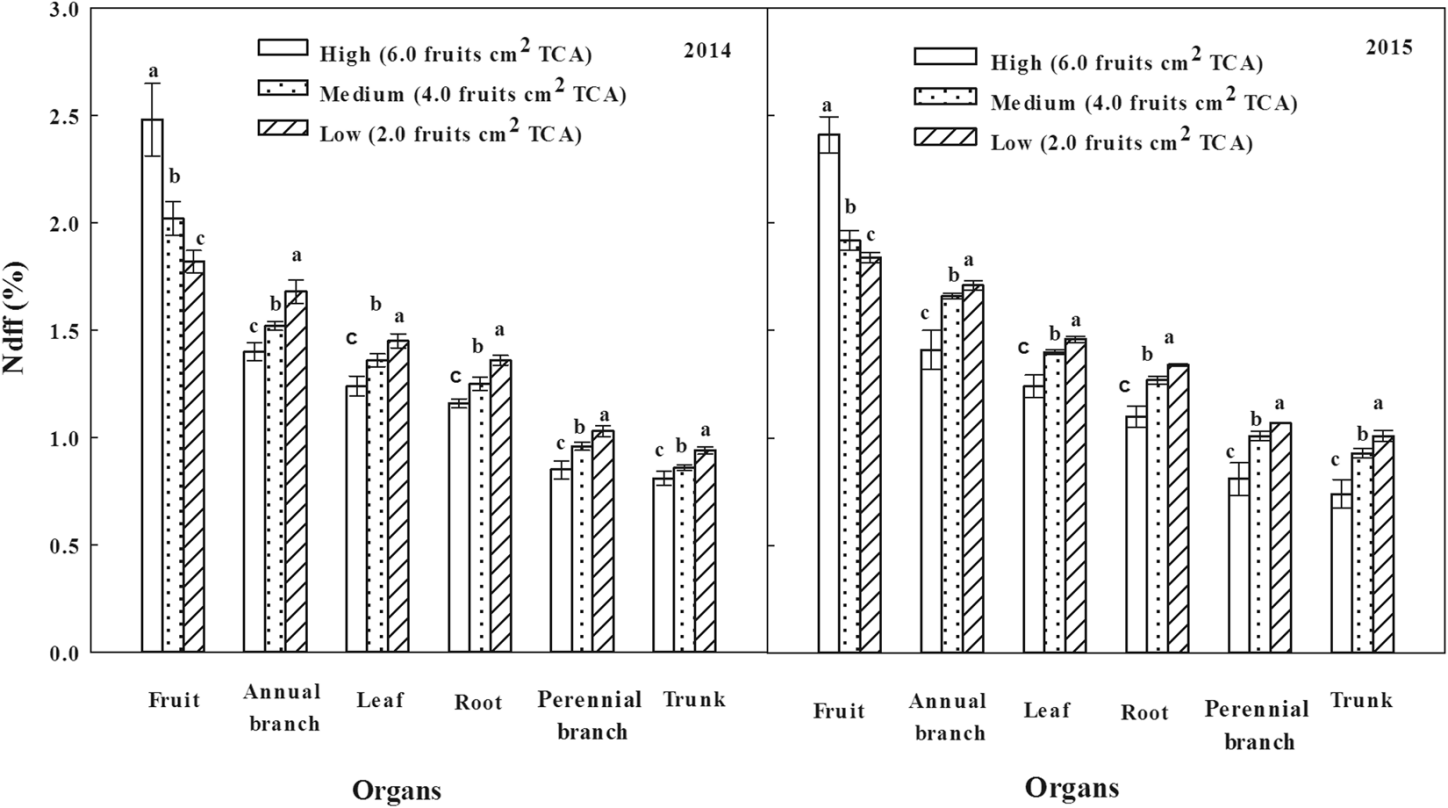
Effects of different crop load treatments on Ndff at the fruit maturity stage in 2014 and 2015.The vertical bar indicates the standard error of the mean.

The Ndff values of organs in different crop load treatments were consistent at the fruit maturity stage in both years, among which the fruits were the largest, and then in the annual branches, leaves, roots and perennial branch, and the trunk was the least (Fig. 1). The Ndff values of annual branches, leaves, roots, trunk and perennial branch on Low-crop load treatment were the largest, followed by the Medium-crop load treatment, and the lowest was at the High-crop load treatment. However, the Ndff values of fruits on the High-crop load treatment were the largest, followed by the Medium-crop load treatment, and the lowest was at the Low-crop load treatment. The research results showed that the fruits were the growth center at the fruit maturity stage, so the most competitive fertilizer ^15^N which was mainly distributed to the fruits. With the decrease of crop load, the capability of absorbing and regulating ^15^N by fruits decreased, but the capability of leaves, the annual branches, other vegetative organs and the storage organs were enhanced.

### The total nitrogen content of plant and ^15^N-urea utilization rate

The total nitrogen content of leaves, annual branches and roots on Low-crop load treatment was the largest at the fruit maturity stage in both years, followed by the Medium-crop load treatment, and the lowest was at the High-crop load treatment (Table 2). However, the total nitrogen content of fruits on the High-crop load treatment was the largest, followed by the Medium-crop load treatment, and the lowest existed at the Low-crop load treatment, but no significant differences were observed in the perennial branches and the trunk. At the fruit maturity stage, the total N content of per tree and ^15^N-urea utilization rate were significantly affected by fruit crop load (Table 2). The total N in plants from Medium and Low-load treatments increased by 6.15% and 38.62%, and 12.21% and 29.55% in 2014 and 2015 respectively, and were significantly higher than from High-load treatment. The plant ^15^N-urea utilization rate on Medium and Low-crop load treatments in 2014 was 1.12 and 1.33 times larger than that of High-crop load treatment, respectively, and it was 1.14 and 1.35 times larger in 2015. The results showed that the capability of plants to absorb ^15^N-urea and the nitrogen utilization rate increased with the decline of the crop load.

**Table 2:** Effects of different crop load treatments on the total N content and ^15^N-urea utilization rate at the fruit maturity stage in 2014 and 2015.

Year	Treatment	Total N content of each organ						Total N content of plant (g)	^15^N-urea utilization rate (%)
		Leaf (g)	Annual Branch (g)	Trunk (g)	Perennial Branch (g)	Fruit (g)	Root (g)		
2014	High-crop load (6.0 fruits cm^−2^ TCA)	13.47 ± 0.2219 c	5.87 ± 0.1587 c	7.89 ± 1.4747 a	10.17 ± 1.8967 a	12.83 ± 0.8998 a	15.61 ± 1.4714 c	65.85 ± 1.6352 c	19.59 ± 1.048 c
	Medium-crop load (4.0 fruits cm^−2^ TCA)	16.76 ± 1.0372 b	7.52 ± 0.7860 b	7.16 ± 0.4469 a	6.88 ± 1.3727 a	8.90 ± 0.5839 b	23.72 ± 1.7336 b	70.96 ± 1.4835 b	22.01 ± 0.7972 b
	Low-crop load (2.0 fruits cm^−2^ TCA)	24.23 ± 1.4333 a	12.08 ± 0.3597 a	7.04 ± 1.0218 a	7.06 ± 1.7182 a	5.07 ± 0.6042 c	37.17 ± 1.8348 a	92.67 ± 2.3962 a	26.13 ± 0.6988 a
2015	High (6.0 fruits cm^−2^ TCA)	13.28 ± 1.1077 c	6.46 ± 0.8729 c	7.87 ± 0.4574a	11.86 ± 0.2158 a	16.04 ± 0.8087 a	16.99 ± 0.9778 c	72.51 ± 3.104 c	21.43 ± 1.3165 c
	Medium (4.0 fruits cm^−2^ TCA)	19.18 ± 1.3471 b	9.59 ± 0.4537 b	7.54 ± 1.0635 a	9.28 ± 0.2754 b	11.78 ± 1.0542 b	23.97 ± 0.7209 b	81.36 ± 1.4749 b	24.46 ± 0.3624 b
	Low (2.0 fruits cm^−2^ TCA)	25.27 ± 0.4875 a	12.65 ± 0.7921 a	7.58 ± 0.1044 a	7.57 ± 1.5409 b	6.96 ± 1.1297 c	33.90 ± 0.8263 a	93.94 ± 2.0547 a	28.95 ± 0.2001 a

Note: Means followed by similar letter within each column are not significantly different at the 0.05 level.

### The ^15^N and ^13^C partitioning rate

**Figure 2:**
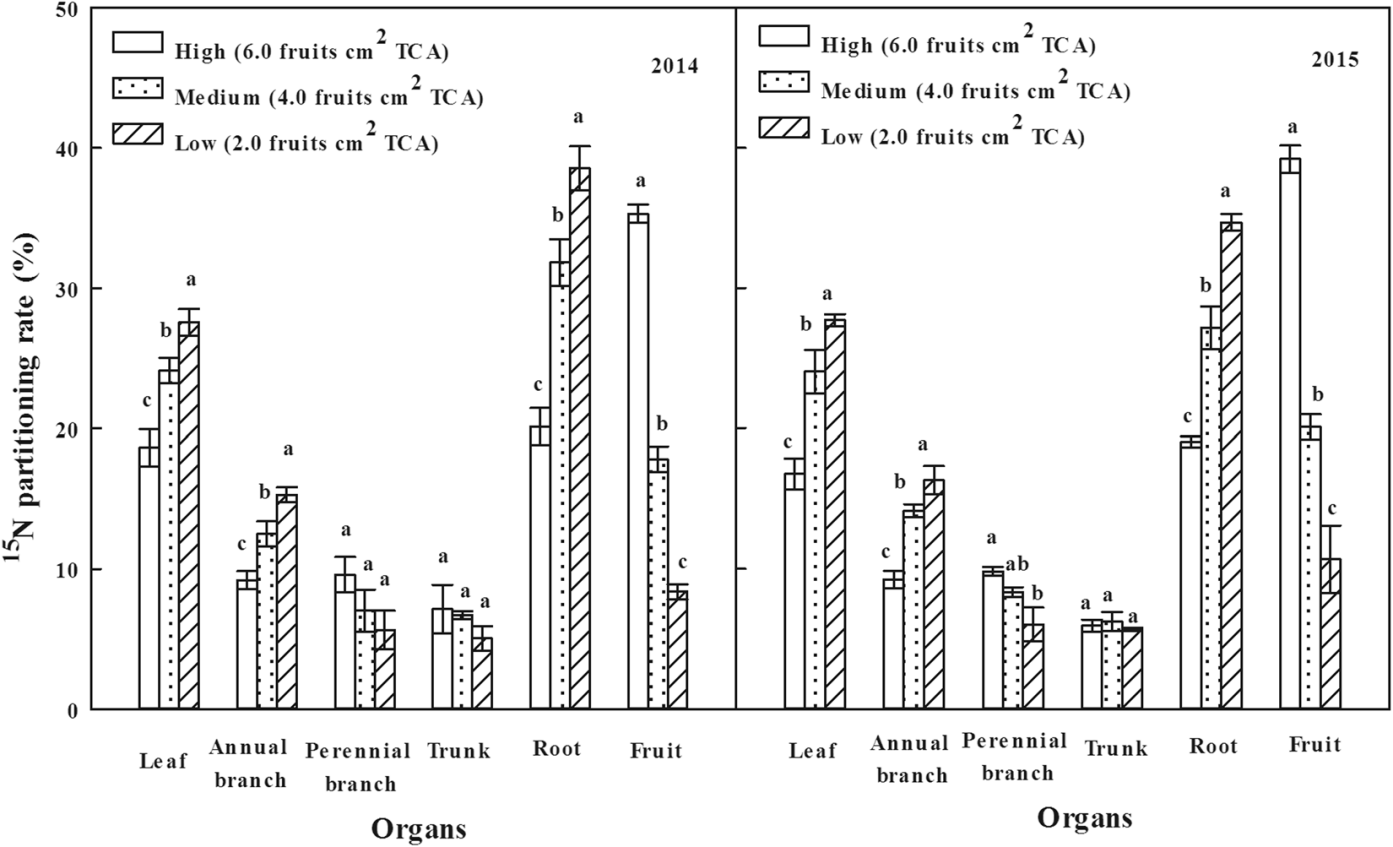
Effects of different crop load treatments on ^15^N Partitioning rate at the fruit maturity stage in 2014 and 2015 (^15^N partitioning rate refers to the ratio of ^15^N absorbed by each organ from fertilizer to ^15^N absorbed by plant from fertilizer). The vertical bar indicates the standard error of the mean.

The percentage of ^15^N in each organ accounted for the total ^15^N content in all organs reflected the distribution of nitrogen fertilizer in the trees and the migration regularity in organs. It showed that ^15^N on High-crop load treatment mainly distributed into the fruit at the fruit maturity stage in both years (Fig. 2), and it was followed by roots, leaves, branches and other organs. With the reduction of crop load, the distribution rate of ^15^N in fruits gradually decreased but it increased in the roots, leaves, annual branches and other organs. The results indicated that the distribution content of nitrogen, which was absorbed by the plant to the reproductive organs, reduced with the decrease of crop load, but its distribution to other organs increased, thereby increasing the storage of the tree nutrition.

**Figure 3:**
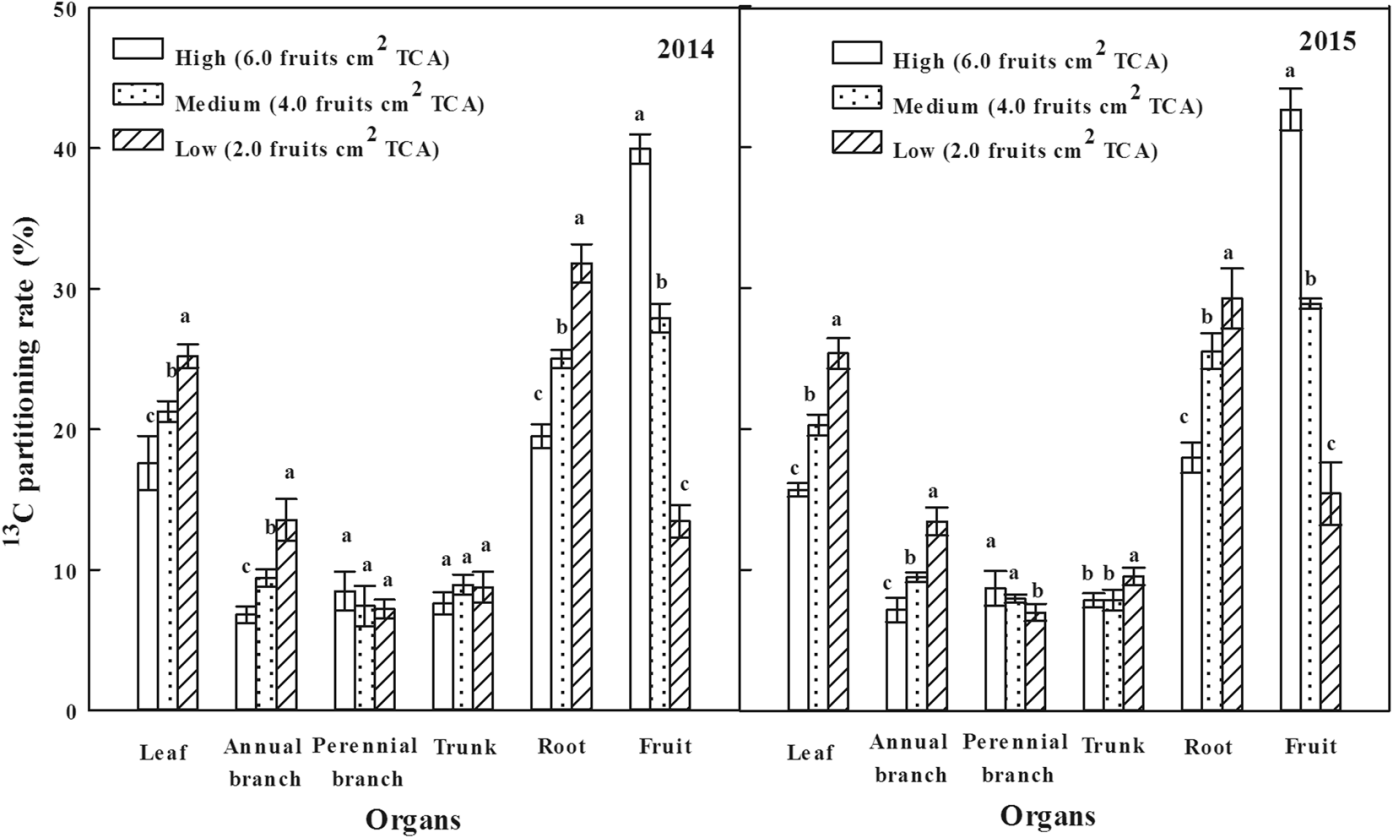
Effects of different crop load treatments on ^13^C Partitioning rateat the fruit maturity stage in 2014 and 2015 (^13^C partitioning rate refers to the ratio of ^13^C content of each organ to the amount of net absorption of ^13^C by plant).

The proportion of ^13^C assimilates assigned to each organ is related to its competitive ability, which referred to the ability of absorbing ^13^C from the labeled leaves in the active parts of metabolism and growth. It was showed that ^13^C in High-crop load treatment was mainly distributed in the fruits at the fruit maturity stage in both years (Fig. 3), and followed by roots, leaves, branches and other organs. With the decrease of crop load, the distribution rate of ^13^C in the fruits gradually reduced, but increased in roots, leaves, annual branches and other organs. The difference of ^13^C distribution rate between perennial branch and trunk were not significant. The results showed that the transportation and distribution of carbohydrates to the fruits decreased but the distribution to the roots, leaves, annual branches and other organs increased with the decrease of crop load, so as to promote the plant vegetative growth.

### Apple yield and quality and economic benefit

The mean fruit weight significantly increased with the decrease of crop load, but fruit yield of each tree significantly decreased at the fruit maturity stage in both years (Table 3). Compared with High-crop load treatment, the mean fruit weight with the treatments of Medium and Low-crop load increased by 15.67% and 34.30% at the fruit maturity stage in 2014, and the values were 14.15% and 32.29% respectively in 2015. Conversely, the yield of each tree with the treatments of Medium and Low-crop load decreased by 25.14% and 54.87% in 2014, and decreased by 21.62% and 53.49% respectively in 2015.

**Table 3:** Effects of different crop load treatments on fruit yield and quality at the fruit maturity stage in 2014 and 2015.

Year	Treatment	Average yield per plant (kg)	Mean fruit weight (g)	Soluble solid (%)	Hardness (Kg /cm^2^)	Soluble sugar (%)	Titratable acids (%)	acid-sugar ratio
2014	High-crop load (6.0 fruits cm^−2^ TCA)	28.12 ± 0.6245 a	236.31 ± 4.4819 c	13.70 ± 0.1682 c	7.33 ± 0.1528 c	12.52 ± 0.2011 c	0.4790 ± 0.0060 c	26.13 ± 0.3451c
	Medium-crop load (4.0 fruits cm^−2^ TCA)	21.05 ± 0.4892 b	273.34 ± 5.4101 b	15.67 ± 0.2757 b	7.80 ± 0.1004 b	13.47 ± 0.1950 b	0.4916 ± 0.0014 b	27.41 ± 0.1914 b
	Low-crop load (2.0 fruits cm^−2^ TCA)	12.69 ± 0.2654 c	317.37 ± 7.5219 a	16.66 ± 0.2707 a	8.33 ± 0.1528 a	14.23 ± 0.1976a	0.5021 ± 0.0058 a	28.35 ± 0.0598 a
2015	High-crop load (6.0 fruits cm^−2^ TCA)	29.05 ± 0.7987 a	243.24 ± 6.2654 c	13.49 ± 0.0862 c	6.90 ± 0.1528 c	12.83 ± 0.4715 c	0.4715 ± 0.1054 c	27.21 ± 0.2317 c
	Medium-crop load (4.0 fruits cm^−2^ TCA)	22.77 ± 0.3027 b	277.67 ± 4.4364 b	15.95 ± 0.0709 b	7.57 ± 0.2487 b	13.78 ± 0.4852 b	0.4852 ± 0.0643 b	28.41 ± 0.1858 b
	Low-crop load (2.0 fruits cm^−2^ TCA)	13.51 ± 0.2523 c	321.78 ± 5.2364 a	16.46 ± 0.1153 a	8.17 ± 0.2082 a	14.63 ± 0.5054 a	0.5054 ± 0.0902 a	28.95 ± 0.2058 a

Note: Means followed by similar letter within each column are not significantly different at the 0.05 level.

Fruit quality appeared to be significantly improved by thinning fruit compared with High-crop load treatment (Table 3). Based on measurements of the soluble solids, hardness, soluble sugar and titratable acid, the differences between the Medium and Low-crop load treatments were obviously significant and both were remarkably higher than the High-crop load treatment. The ratio of sugar to acid and their contents can influence apple flavour. The ratio of sugar to acid from Medium and Low-load treatments increased by 1.28% and 2.22%, and 1.20% and 1.74% in 2014 and 2015 respectively, and were significantly higher than from High-load treatment. Thus, it showed that the treatments of Medium and Low-crop load significantly improved the fruit quality at the fruit maturity stage.

With the reduction of fruit crop load, the fruit yields and total income of orchard decreased. However, the fruit quality was improved, leading to higher average apple prices and lower mean labor costs and service charges, which greatly reduced the cost of production (Tables 3 and 4). Hence, mean annual net profits of Medium and Low-crop load treatments increased by 19.39% and 2.63% compared with High-crop load treatment, respectively (Table 4).

**Table 4:** Mean annual gross revenue, cost, and net profit for apple production under different crop load treatments.

Treatment	Total revenue	Fertilizer costs	Labor cost	Other costs	Net profit	% change relative to ck
($ ha^−1^ year^−1^)						
High-crop load (6.0 fruits cm^−2^ TCA)	35536.1	5622.2	9220.4	2698.6	17994.9	—
Medium-crop load (4.0 fruits cm^−2^ TCA)	37401.8	5622.2	7596.7	2698.6	21484.3	19.39
Low-crop load (2.0 fruits cm^−2^ TCA)	31961.0	5622.2	5172.5	2698.6	18467.7	2.63

Note: Average apple prices in China: High-crop load $750.0 t^−1^, Medium-crop load $1050.0t^−1^, Low-crop load $1500.0t^−1^. Labor costs included apple bagging and removing, picking and fertilizing. Other costs included irrigation, pesticides, insecticides, pruning, other materials and expenses.

## Discussion

The leaf is the main organ of photosynthesis to produce the dry matter, and the chlorophyll is the main chemical compounds with nitrogen^38,39^. The increasing leaf area and chlorophyll content in the late growth stage are propitious to improve photosynthesis and delay leaf senescence^24,40^. Nii (1997) suggested that increasing crop load resulted in a decrease in leaf area, dry mass of each unit leaf area, and an increase in chlorophyll content^41^. Wunsche *et al*. (2005) also pointed out that increased crop load could increase chlorophyll concentration^42^. In the present study, the leaf area on Medium and Low-crop load treatments were significantly higher than that of High-crop load treatment (Table 1), which was consistent with the above results, but the leaf chlorophyll content and photosynthetic rate reduced with the increase of fruit crop load (Table 1), which were contrary to the previous studies. The reason is that the competition for the photosynthetic nutrient in the late development stage of fruit exists between shoots and roots, and the photosynthetic products are mainly transported to the “sink” organ (fruit) with the increasing crop load. Thus, the roots will appear “hunger” due to the insufficient organic nutrient^43^, inhibiting the absorption of mineral nutrition and water. This phenomenon will lead to the decrease of the nutrient level in the leaves that might be the main cause of decrease of photosynthetic rate^44^. In addition, the nutrient content of leaves at later growth stage showed that the total nitrogen content in the leaves with the Medium and Low-crop load treatments was significantly higher than that of High-crop load treatment (Table 1), which also showed that thinning fruit could significantly improve the nitrogen content of leaves, so as to increase the chlorophyll content of leaves.

This experiment with ^15^N tracer technique indicated that the utilization rate of ^15^N on Medium and Low-crop load treatments were significantly higher than that of High-crop load treatment, increased by 1.12–1.33 times and 1.14–1.35 times in 2014 and 2015, respectively (Table 2). This is related to the obviously higher Ndff value of roots on Medium and Low-crop load treatments than that on High-crop load treatment, which enhanced the absorption capability of roots for nitrogen (Fig. 1). And, this conclusion was proved by the obvious higher total nitrogen content in other organs (Table 2) and the root-shoot ratio (Table 1) of Medium and Low-crop load treatments than those of High-crop load treatment. Fruit tree mineral nutrition and fertilization had significant effects on the fruit yields^16^, and an optimal nitrogen supply was of great importance in order to avoid negative effects such as low fruit quality^20,45^ and storage ability^13,46^. However, in Chinese high yield orchards, the fruit growers often provide a large amount of nitrogen fertilizer to the trees to ensure higher yield. In the present study, the high amount of crop load did not promote the absorption and utilization of nitrogen, which was mainly caused by the decrease of carbohydrate in roots provided by the shoot to affect the activities of plant root and then to limit the absorption and utilization of nitrogen. This is an important reason for the high amount of nitrogen fertilizer but the low utilization rate of nitrogen in current apple orchards in China. Besides, an excessive nitrogen fertilizer supply will not only increase production costs, but also cause the pollution of the environment. Therefore, it is of critical importance to supply appropriate amount of the nutrients to the trees especially on high fruit crop load.

The carbohydrates produced by the leaves followed the allocation principle of priority to growth centers of plant^47^. The fruits were the center of trees at the fruit maturity stage, especially for perennial fruit trees, the contradiction of the “source” and “sink” was prominent because of the competition for the photosynthetic nutrition between roots and shoots. Thus, the increasing crop load could aggravate the competition for the carbohydrate and reduce the nutrition stored in the trees, which was not conducive to improve the cold resistance and construction of new organs, fruit yield and quality in the next year^46^. The distribution rate of ^13^C and ^15^N in this research showed that reproductive organs’ (fruits) ability of competition for ^13^C and ^15^N was the strongest at the fruit maturity stage, and ^13^C and ^15^N in plants mainly allocated to the reproductive organs (Figs 2 and 3). However, the content of ^13^C and ^15^N allocating to the reproductive organs gradually decreased with the decline of the crop load, but the content allocating to the vegetative organs (annual branches and leaves) and storage organs (roots and perennial branches) increased, so as to promote the vegetative growth and increase the storage nutrition of the trees. It was also proved by the higher total nitrogen content in nutrient organs (annual branches and leaves) and storage organs (roots) on Medium and Low-crop load treatments than that of High-crop load treatment. Thus, the appropriate fruit thinning reduced the consumption of carbon and nitrogen nutrient, promoted the current vegetative growth and also increased the reserve of carbon and nitrogen in the vegetative organs. This was conducive to the supply of plant’s growth in the spring of the next year and laid the foundation for next year’s harvest.

Solomakhin and Blanke (2010) showed that thinning fruit could increase the mean fruit weight and improve fruit quality^48^. Goffinet (1995) believed that the thinning fruit could stimulate fruit enlargement^19^, and the increasing size of fruit was related to the promotion of cell division and expansion at the same time and the increasing amount and size of cells^49^. However, in the production, orchard owners have focused on yield and ignored quality by little or no fruit thinning, thereby resulting in the decrease of the proportion of good quality fruits. The present study showed that the Medium and Low-crop load treatments significantly improved the fruit quality (Table 3). These results are similar to those previously observed^17,50^, showing improved apple quality in terms of each fruit weight, size, and firmness with a decrease in crop load of each tree^10,17,51^. The significant increase in the rate of good quality fruit resulted in overall economic benefits. Economic benefits in the fruit industry were mainly determined by the cost of production, fruit yield and quality, as well as other determinants^52^. The present study suggested that with the reduction of fruit crop load, the mean labor costs and service charges were also reduced, greatly reducing the cost of production. Compared with High-crop load treatment, the average annual net profit of Medium and Low-crop load treatments increased by 19.39% and 2.63% (Table 4), among which the Medium-crop load treatment was the highest, and it could not only guarantee the mean fruit weight and improve the fruit quality, but also did not notably reduce the yield of apple.

In summary, the carbon and nitrogen nutrition in mature M.26 interstocks apple orchards were distributed reasonably after the thinning fruit treatments on Medium and Low-crop load, and the utilization rate of nitrogen fertilizer was improved significantly. Besides, thinning fruit obviously increased fruit quality and the reserve of storing nutrient in storage organs. The comprehensive benefits on Medium-crop load level were the best (4.0 fruits cm^−2^ TCA), which could not only ensure the fruit yield and quality, but also could store more nutrients to supply construction of new organs and flower bud differentiation for the trees in the following year. In the production, in addition to taking a reasonable fruit crop load, the comprehensive management measures must be strengthened, including soil water, pruning, flower and fruit management, pest control etc., to ensure the high quality and stable yield of fruit trees.

## Methods

### Experimental sites and materials

Field experiments were performed from 2014 to 2015 in an apple orchard at Laishan, Yantai City, Shandong Province, Northeast China (121^◦^43′00′′E, 37°50′47′′N). The climate is classified as semi-humid, with annual average precipitation of 672.5 mm, of which nearly 70% occurs from June to September. The annual mean temperature (1984–2015) is 12.5 °C, and there are about 210 frost-free days each year.

Trees were planted in the year 2008 in rows spaced 1.5 m apart with 4 m between the rows and trained as a slender spindle. The commercially important apple (Malus × *domestica* Borkh.) cultivar ‘Red Fuji’ was grafted on the dwarfing interstock M.26, and then was grafted on the rootstock *Malus hupehensis* Rehd (‘Red Fuji’/M.26/*Malus hupehensis* Rehd). The soil was brown loam with pH 5.18, soil organic matter content was 7.66 g ·kg^−1^, NO_3_^−^-N, NH_4_^+^-N, available P and available K was 25.14, 14.26, 34.12 and 221.32 mg ·kg^−1^, respectively.

### Experimental design

Trees with identical crop loads and development attributes were selected and marked for the crop load treatments. Before thinning, the crop loads had been calculated based on the numbers of fruits per trunk cross-sectional area (cm^2^, TCA) of the trees. In the present study, 18 plants were selected and divided into 3 crop load treatments. Each treatment was divided into 2 groups with 3 replicates per group. Treatment 1: High-crop load (6.0 fruits cm^−2^ TCA), Treatment 2: Medium-crop load (4.0 fruits cm^−2^ TCA). Treatment 3: Low-crop load (2.0 fruits cm^−2^ TCA). Fruit thinning was carried out manually after 30 days of blossom in 2014 and 2015, respectively. Group 1: After fruit thinning (May 20th), the treatment of ^15^N labeling was performed. Ten grams of ^15^N-urea (CO(^15^NH_2_)_2_, produced by Shanghai Research Institute of Chemical Industry: abundance of 10.14%), 190 g of normal urea CO(NH_2_)_2_, 210 g of ammonium phosphate ((NH_4_)_2_HPO_4_) and 120 g of potassium sulfate (K_2_SO_4_) were mixed and applied to the soil for each tree. ^13^C pulse labeling was performed at the fruit maturity stage (October 10th). Group 2: Each plant was applied with 200 g of normal urea (CO(NH_2_)_2_), 210 g of ammonium phosphate ((NH_4_)_2_HPO_4_) and 120 g of potassium sulfate (K_2_SO_4_) as control. The method of fertilization was digging a ring trench from the center of 30 cm whose depth and width were about 20 cm per tree. The growth conditions, cultivation and management of each treatment were consistent.

Group 1: ^13^C pulse labeling treatment was carried out in a labeling chamber with transparent agricultural film at the fruit maturity stage (October 10th) in 2014 and 2015, respectively. The whole plant was covered and sealed by the labeling chamber, and checked the seal of the labeling chamber before labeling. One end of a hollow tube was put on a balloon and the other end with a rubber pipette bulb. According to the inflated state of the balloon, we could determine whether the chamber was well-sealed. Before sealing the labeling room, ten grams of Ba ^13^CO_3_ (^13^C abundance is 98%, the proportion of ^13^C in all carbon elements) was put into a beaker and iron powder was reduced into the labeling room. Labeling work was started at 9:00 am (October 10th), and the beaker with Ba ^13^CO_3_ was injected into the certain HCl of 1 mol·L^−1^ with a syringe. Hydrochloric acid was injected into the beaker every 0.5 h for 4 h in order to maintain the concentration of CO_2_.The plants were destructively sampled after 72 h (at 9:00 am on October 13th). At the same time, another group of control plants was used as a blank of ^13^C labeling (natural abundance of ^13^C).

### Measurement of chlorophyll content, photosynthetic rate and leaf area

The middle leaves of new shoots were taken to analyze net photosynthetic rate (Pn) which was measured with a LI-6400XT portablephoto synthesis system (LI-Cor, Lincoln, NE, USA) from 9:00 to 11:00 am under the standardized climatic condition at the fruit maturity stage in 2014 and 2015, respectively. Meanwhile, the leaf area was measured with a leaf area meter (YMJ-B; Minolta, Tokyo, Japan), and the chlorophyll content (SPAD value) was measured with a chlorophyll meter (SPAD-502; Minolta, Tokyo, Japan).

### Fruit yield and quality

Yield (kg per tree) was evaluated at the fruit maturity stage. Meanwhile, 10 apples were picked from each trial plant to measure the fruit weight and quality. The content of soluble sugar and titratable acid were measured by the method of Anthrone colorimetry and NaOH titration, respectively. The content of soluble solids was determined by saccharometer and the hardness was determined by HP-230 hardness tester^54^.

### ^15^N and ^13^C

Destructive sampling was applied for the entire plant with ^13^C after 72 h of labeling (at 9:00 am on October 13th). All the trial plants were subjected to destructive sampling, and the whole plant samples were divided into leaves, the annual branches, the perennial branches, the central stems, the roots and the fruits. The samples were washed by branch water, detergent, branch water and 1% hydrochloric acid in order, and then with deionized water for 3 times. The samples were then dried at 80 °C, followed with homogenization by electric grinder and filtration with 0.25 mm mesh screen. The samples of Group 1 were used to determine the content of nitrogen and the abundance of ^15^N and ^13^C, and those of Group 2 were used to determine the nature abundance of ^13^C as a blank control of the corresponding organs of crop fruit treatments of Group1, respectively. The content of nitrogen was determined by the method of Kjeldahl measurement, and the abundance of ^15^N was measured with ZHT-03 mass spectrometer made in Beijing analytical instrument factory (Chinese Academy of Agricultural Sciences). The abundance of ^13^C was measured with DELTAV^plus^XP advantage isotope ratio mass spectrometer analyzed by China Academy of Forestry Sciences Stable Isotope Laboratory.

**Calculation of** ^**15**^ **N**

1 $${\rm{Ndff}}\,( \% )\,=\frac{{\rm{abundance}}\,{\rm{of}}\,{}^{{\rm{15}}}{\rm{N}}\,{\rm{in}}\,{\rm{plant}}-{\rm{natural}}\,{\rm{abundance}}\,{\rm{of}}\,{}^{{\rm{15}}}{\rm{N}}}{{\rm{abundance}}\,{\rm{of}}\,{}^{{\rm{15}}}{\rm{N}}\,{\rm{in}}\,{\rm{fertilizer}}-{\rm{natural}}\,{\rm{abundance}}\,{\rm{of}}\,{}^{{\rm{15}}}{\rm{N}}}\times 100 \% $$

2 $${}^{{\rm{15}}}{\rm{N}}\,{\rm{utilization}}\,{\rm{rate}}\,( \% )=\frac{{\rm{Ndff}}\times {\rm{total}}\,{}^{{\rm{15}}}{\rm{N}}\,{\rm{of}}\,{\rm{organs}}\,({\rm{g}})\,}{{}^{{\rm{15}}}{\rm{N}}\,{\rm{fertilization}}\,({\rm{g}})\,}\times 100 \% $$

3 $${}^{{\rm{15}}}{\rm{N}}\,{\rm{partitioning}}\,{\rm{rate}}\,( \% )=\frac{{}^{{\rm{15}}}{\rm{N}}\,{\rm{absorbed}}\,{\rm{by}}\,{\rm{each}}\,{\rm{organ}}\,{\rm{from}}\,{\rm{fertilizer}}\,({\rm{g}})}{{\rm{total}}\,{}^{{\rm{15}}}{\rm{N}}\,{\rm{absorbed}}\,{\rm{by}}\,{\rm{plant}}\,{\rm{from}}\,{\rm{fertilizer}}\,({\rm{g}})}\times 100 \% $$

**Calculation of** ^**13**^ **C**

4 $$\begin{array}{c}{\rm{Abundance}}\,{\rm{of}}\,{}^{13}{\rm{C}}:\,{{\rm{F}}}_{{\rm{i}}}( \% )=\frac{({\rm{\delta }}{}^{13}{\rm{C}}+1000)\times {{\rm{R}}}_{{\rm{PBD}}}}{({\rm{\delta }}{}^{13}{\rm{C}}+1000)\times {{\rm{R}}}_{{\rm{PBD}}}+1000}\times 100 \% \\ {{\rm{R}}}_{{\rm{PBD}}}({\rm{standard}}\,{\rm{ratio}}\,{\rm{of}}\,{\rm{carbon}}\,{\rm{isotope}})=0.0112372\end{array}$$

5 $${\rm{Carbon}}\,{\rm{content}}\,{\rm{of}}\,{\rm{each}}\,{\mathrm{organ}:C}_{{\rm{i}}}={\rm{amount}}\,{\rm{of}}\,{\rm{dry}}\,{\rm{matter}}\,({\rm{g}})\times {\rm{total}}\,{\rm{carbon}}\,{\rm{content}}\,( \% )$$

6 $$\begin{array}{c}{\rm{Content}}\,{\rm{of}}\,{}^{13}{\rm{C}}\,{\rm{of}}\,{\rm{each}}\,{\rm{organ}}:{}^{13}{\rm{C}}_{i}({\rm{mg}})=\frac{{{\rm{C}}}_{{\rm{i}}}\times ({{\rm{F}}}_{i}-{{\rm{F}}}_{nl})}{100}\times 1000\\ {{\rm{F}}}_{nl}:{\rm{no}}\,{}^{13}{\rm{C}}\,\mathrm{labeling},\,{\rm{natural}}\,{\rm{abundance}}\,{\rm{of}}\,{}^{13}{\rm{C}}\,{\rm{of}}\,{\rm{each}}\,{\rm{organ}}\end{array}$$

7 $${}^{13}{\rm{C}}\,{\rm{partitioning}}\,{\rm{rate}}:\,{}^{13}{\rm{C}}\,( \% )=\frac{{}^{13}{\rm{C}}_{i}}{{}^{13}{\rm{C}}_{{\rm{net}}\,{\rm{absorption}}}}\times \mathrm{100} \% $$

### Data statistical analysis

Microsoft Excel 2003 was used for data processing, and Sigma Plot 12.2 helped with drawing figures. The single factor variance was analyzed with DPS 7.05 software and the significance of difference was calculated with LSD method, with the significant level of α = 0.05^55^.
